# Mechanisms Underpinning Adaptations in Placental Calcium Transport in Normal Mice and Those With Fetal Growth Restriction

**DOI:** 10.3389/fendo.2018.00671

**Published:** 2018-11-20

**Authors:** Christina E. Hayward, Kirsty R. McIntyre, Colin P. Sibley, Susan L. Greenwood, Mark R. Dilworth

**Affiliations:** ^1^Maternal and Fetal Health Research Centre, Division of Developmental Biology and Medicine, Faculty of Biology, Medicine and Health, School of Medical Sciences, University of Manchester, Manchester, United Kingdom; ^2^Manchester Academic Health Science Centre, St. Mary's Hospital, Manchester University NHS Foundation Trust, Manchester, United Kingdom

**Keywords:** placenta, calcium, adaptation, FGR, IUGR, mouse

## Abstract

Fetal delivery of calcium, via the placenta, is crucial for appropriate skeletal mineralization. We have previously demonstrated that maternofetal calcium transport, per gram placenta, is increased in the placental specific insulin-like growth factor 2 knockout mouse (P0) model of fetal growth restriction (FGR) compared to wild type littermates (WTL). This effect was mirrored in wild-type (WT) mice comparing lightest vs. heaviest (LvH) placentas in a litter. In both models increased placental calcium transport was associated with normalization of fetal calcium content. Despite this adaptation being observed in small normal (WT), and small dysfunctional (P0) placentas, mechanisms underpinning these changes remain unknown. Parathyroid hormone-related protein (PTHrP), elevated in cord blood in FGR and known to stimulate plasma membrane calcium ATPase, might be important. We hypothesized that PTHrP expression would be increased in LvH WT placentas, and in P0 vs. WTL. We used calcium pathway-focused PCR arrays to assess whether mechanisms underpinning these adaptations in LvH WT placentas, and in P0 vs. WTL, were similar. PTHrP protein expression was not different between LvH WT placentas at E18.5 but trended toward increased expression (139%; *P* = 0.06) in P0 vs. WTL. PCR arrays demonstrated that four genes were differentially expressed in LvH WT placentas including increased expression of the calcium-binding protein calmodulin 1 (1.6-fold; *P* < 0.05). Twenty-four genes were differentially expressed in placentas of P0 vs. WTL; significant reductions were observed in expression of S100 calcium binding protein G (2-fold; *P* < 0.01), parathyroid hormone 1 receptor (1.7-fold; *P* < 0.01) and PTHrP (2-fold; *P* < 0.05), whilst serum/glucocorticoid-regulated kinase 1 (SGK1), a regulator of nutrient transporters, was increased (1.4 fold; *P* < 0.05). Tartrate resistant acid phosphatase 5 (TRAP5 encoded by Acp5) was reduced in placentas of both LvH WT and P0 vs. WTL (1.6- and 1.7-fold, respectively; *P* < 0.05). Signaling events underpinning adaptations in calcium transport are distinct between LvH placentas of WT mice and those in P0 vs. WTL. Calcium binding proteins appear important in functional adaptations in the former whilst PTHrP and SGK1 are also implicated in the latter. These data facilitate understanding of mechanisms underpinning placental calcium transport adaptation in normal and growth restricted fetuses.

## Introduction

Placental dysfunction, associated with reduced rates of nutrient uptake (1–3), is a major cause of fetal growth restriction (FGR), the failure of a fetus to reach its growth potential (4). FGR is a significant risk factor for stillbirth (5, 6). Additionally, FGR infants demonstrate increased incidence of childhood diseases such as cerebral palsy, and adulthood diseases including heart disease, stroke, diabetes and osteoporosis (7–11). The current lack of therapies for FGR (12) emphasizes the need for better understanding of how fetal development is normally achieved and how it is dysregulated in FGR.

Placental transfer of calcium increases over gestation to match fetal demand and ensure appropriate fetal skeletal mineralization (13). Poor fetal provision of calcium *in utero* has been linked with an increased risk of developing osteoporosis later in life (14). Maternofetal transfer of calcium across the placenta involves calcium moving from maternal blood into the syncytiotrophoblast (transporting epithelium of the placenta) down an electrochemical gradient through calcium permeable cation channels (e.g., TRPV6, transient receptor potential vanilloid type 6) on the maternal-facing microvillous membrane (MVM) (15–17). Once in the trophoblast cytosol calcium is buffered to avoid overly increasing the intracellular concentration, and shuttled to the fetal-facing basal membrane (BM) by calcium binding proteins such as calbindin-D_9K_ (16). Calcium is actively transported across the BM by plasma membrane calcium ATPases (PMCA) into the fetal compartment. The actions of PMCA help to maintain calcium concentrations in the fetus above those found in maternal blood. Unlike the activity of other nutrient, and especially amino acid, transport systems that are reduced in placentas of growth restricted fetuses (1–3), the activity of PMCA is increased in human FGR (18) as is the maternofetal transfer of calcium in a rodent model of FGR, the placental-specific insulin-like growth factor *2* (P0) knockout mouse (19).

Optimal fetal growth depends on adequate nutrient delivery and placental supply can be adapted to meet the metabolic needs of the developing fetus. In pregnancies with normal outcomes, adaptation of placental transport in relation to placental size appears important in both women and mice (20–22). Our previous studies of wild-type (WT) mouse litters demonstrated that maternofetal calcium transfer across the lightest placentas is adaptively up-regulated, compared to the heaviest placentas, so that all fetuses, whether with relatively lighter or heavier placentas, accrue an appropriate level of calcium relative to their size near term (22). We suggested that this increased maternofetal transfer of calcium (per gram placenta), which coincides with increased placental calbindin-D_9K_ expression at embryonic day (E)18.5, is an example of a placental adaptation that promotes fetal calcium acquisition despite a relatively small placental size. We also found a normalization of fetal calcium accretion by E18.5, following a reduction at E16.5, which may be indicative of a fetus signaling to its placenta, by as yet unknown mechanisms, to increase maternofetal transfer of calcium. The gestational timing of this adaptation in WT mice was similar to that which we previously observed in the P0 knockout mouse (19), and points to a role for fetal nutrient demand in driving this adaptation via altered expression of placental calcium binding proteins. These data showed that placental adaptations are an important feature of both normal and compromised fetal growth and help to ensure appropriate calcium acquisition relative to the size of the fetus.

Nothing is yet known regarding the underlying mechanisms that affect adaptation of placental calcium transport and in particular the fetal and/or placental signals that may be important in this process. Therefore, in this study we investigated mechanisms underlying the adaptive up-regulation of maternofetal calcium transfer. Initially, parathyroid hormone-related protein (PTHrP) was investigated as a candidate fetal signal. PTHrP is produced in a number of tissues including, but not limited to, the placenta, fetal membranes and fetal brain, liver, bone and parathyroid glands (23). In these tissues, there are multiple secretory mature peptides which have a range of different biological functions that can be elicited through endocrine, paracrine, autocrine and intracrine mechanisms [reviewed by (24)]. In women, the concentration of PTHrP in maternal serum, and PTHrP expression in amnion and choriodecidua, are both increased in late gestation in parallel with the rapid increase in fetal growth and calcium accretion (25–27). Previous studies in rodents have demonstrated the importance of PTHrP and its receptor, the PTH/PTHrP receptor, in fetal development. Deletion of these genes in mice results in neonatal death, due to skeletal dysplasia (28) or death *in utero* mid gestation due to growth restriction (29). In a spontaneously hypertensive rat model, inappropriate levels of PTHrP in the placenta, fetal plasma and amniotic fluid were associated with compromised fetal growth (30, 31). Enhancing endogenous levels of PTHrP, by the addition of a PTH/PTHrP receptor antagonist, improves fetal growth in this rat model (31). In PTHrP knockout mice, maternofetal calcium transfer and fetal calcium accretion are increased despite fetal hypocalcaemia and lack of a maternal fetal calcium gradient (32–34). In human pregnancies complicated by FGR, PTHrP expression in fetal membranes and placenta is increased in cases of preterm FGR (35), and concentrations are elevated in cord blood (18). PTHrP also stimulates PMCA activity in BM vesicles isolated from human placenta (36). Thus, we hypothesized that PTHrP is a candidate signal stimulating an increase in calcium transfer and would be elevated in: (1) placental tissue and tissues from fetuses of the lightest vs. heaviest (LvH) placentas in WT mice; and (2) in placentas of P0 fetuses compared to their WT littermates (WTL). Using calcium pathway-focused PCR arrays we also tested the hypothesis that placental mechanisms underpinning the adaptive increase in calcium transfer in WT mice and in P0 mice would be similar.

## Materials and methods

### Animals

Experiments were performed in accordance with the UK Animals (Scientific Procedures) Act of 1986 under the authority of a UK Home office project license (PPLs 40/3385 and P9755892D) and were authorized by the Animal Welfare and Ethical Review Board of the University of Manchester. The methods stated in this study adhere to the ARRIVE guidelines (37) and comply with the animal ethical principles under which the journal operates.

Wild-type C57Bl/6J (Envigo, UK) females (10–16 weeks old) and males (12–26 weeks old) were mated and discovery of a copulation plug was used to define embryonic day (E)0.5 (term = E19.5). Mice were provided with nesting material and communally housed (with the exception of stud males that were individually housed) in individually ventilated cages under a constant 12 h light/dark cycle at 21–23°C with free access to food (Beekay Rat and Mouse diet, Bantin and Kingman) and water (Hydropac, Denver, US). Pregnant female mice were euthanized (cervical dislocation appropriate under ASPA schedule 1) and a laparotomy and hysterotomy performed. All fetuses were rapidly killed by cervical dislocation.

On E18.5 (*N* = 20 litters), pregnant WT females were euthanized and fetuses and placentas were rapidly harvested, blotted and wet weights measured. The lightest (*n* = 20) and heaviest (*n* = 20) placentas were identified in each litter. All placentas and fetuses were snap frozen and stored at −80°C. In 9/20 litters, brains (*n* = 18; 9 from the lightest placental group, 9 from the heaviest) and livers (*n* = 18; 9 from lightest, 9 from heaviest) from fetuses corresponding to the lightest and heaviest placentas were immediately dissected, snap frozen and stored at −80°C. Fetal weight histograms were constructed and a non-linear regression performed (Gaussian distribution) from which individualized fetal weight centiles were calculated as described previously (38).

Placental specific insulin-like growth factor 2 (*Igf2*) (P0) knockout mice (*N* = 10 litters), which had deletion of the U2 exon of the *Igf2* gene, were generated as previously described (39) and were a kind gift from Dr Miguel Constância and Professor Wolf Reik. C57BL/6J female mice (8–14 weeks old) and males heterozygous for the P0 deletion (10–32 weeks old) were mated and produced mixed litters of WTL fetuses and growth restricted fetuses [P0; reported birthweight 78% compared to WTL at E19 equivalent to E18.5 in the current study (40)]. Embryonic day was defined as above. At E18.5, placentas and fetuses (40 WTL; 38 P0 from 10 litters) were weighed, snap frozen and stored at −80°C. Fetal tail tips were collected from all fetuses and stored at −20°C for genotype determination.

The aim of the study, comparing lightest vs. heaviest placentas or those from WTL vs. P0 mice within a single litter, meant that randomization or blinding of the samples was not possible.

### Genotyping of P0 knockout mice

Genotype (WTL or P0) was determined for all fetuses from P0 mice according to a previously published genotyping protocol (41, 19). In brief, genomic DNA was extracted from fetal tail tips using a DNeasy kit (Qiagen, Manchester, UK). *Igf2* P0^+/−^ mutants were identified with a specific primer pair to amplify a 740 bp fragment across the 5 kb deletion (P0 dF 5′-TCCTGTACCTCCTAACTACCAC−3′ and P0 dR 5′-GAGCCAGAAGCAAACT−3′) and a primer to amplify a 495 bp fragment from the WT allele (5′-CAATCTGCTCCTGCCTG−3′). PCR conditions were as follows: 4 min denaturation at 94°C; 35 cycles of 1 min at 94°C, 1 min at 56°C, 1 min at 72°C; and 10 min final extension at 72°C. Samples were loaded with bromophenol blue and run on a 1.5% agarose gel. Bands were visualized using an InGenius transilluminator (Sygene Bio, Cambridge, UK).

### Protein expression

The lightest and heaviest placentas from WT mice (*N* = 7 litters) and placentas of P0 and WTL (*N* = 8 litters, 1 paired P0 and WTL placenta per litter selected at random) were homogenized and processed as described previously (19). Briefly, whole homogenates were separated, by means of centrifugation, into cytosolic fractions. Due to the small amount of starting tissue, whole homogenates of fetal tissues (brains and livers; *N* = 9 litters) were used for protein expression studies.

SDS-PAGE was performed followed by electrotransfer to Immobilon-FL PVDF membranes (Millipore UK Ltd., Watford, UK). Primary antibodies included: rabbit polyclonal antibodies for serum/glucocorticoid-regulated kinase 1 (SGK1; 1 μg/ml; ab43606; Abcam, Cambridge, UK) and calmodulin (CaM; 2 μg/ml; sc-5537; Santa Cruz Biotechnology c/o Insight Biotechnology Ltd, Wembley, UK); rabbit monoclonal antibody for tartrate-resistant acid phosphatase (TRAP; 0.9 μg/ml; ab191406; Abcam); and goat polyclonal antibody for PTHrP (1 μg/ml; N-19, sc-9680; Santa Cruz Biotechnology). β-actin (0.5 μg/ml; ab8227; Abcam) or β-tubulin (0.9 μg/ml; ab6046; Abcam) was used as a loading control; when used no difference was observed in β-actin or β-tubulin expression between groups. Negative controls were by omission of primary antibody. Immunoreactive species were detected with fluorescent-conjugated secondary antibodies (Li-COR Biosciences, Cambridge, UK) and membranes imaged using an Odyssey Sa Infrared Imaging System (Li-COR). Signal density was measured using Image Studio Lite (Li-COR). All signals were in the linear range of detection. Protein expression was compared separately between the lightest and heaviest placentas, fetal tissues from lightest and heaviest placentas, and WT and P0 samples.

### RT^2^ profiler PCR arrays

RNA was extracted from whole placentas (*N* = 7 litters: *n* = 7 lightest, *n* = 7 heaviest; *N* = 6–7 litters: *n* = 6–7 WTL, *n* = 6–7 P0, 1 paired P0 and WTL placenta per litter selected at random) using an RNeasy Mini Kit (74104; Qiagen, Manchester, UK), RNase-Free DNase set (79254; Qiagen) and measured by a Thermo Scientific NanoDrop 2000C spectrophotometer (A_260_/A_280_ range 2.06–2.12). Any contaminating genomic DNA was removed and cDNA was synthesized from 0.5 μg RNA per sample using the RT^2^ first strand kit (330401; Qiagen; genomic DNA elimination mix for 5 min at 42°C, on ice for 1 min; reverse transcription mix for 42°C for 15 min followed by 5 min at 95°C). Expression of 168 related genes, 5 reference genes and quality controls was measured in each placenta using RT^2^ Profiler PCR arrays (PAMM-066Z mouse cAMP/calcium signaling pathway finder and PAMM-170Z mouse osteoporosis array; 96-well format; Qiagen) with RT2 SYBR® Green ROX™ qPCR mastermix (330523; Qiagen) on a Stratagene MX3005P®, according to the manufacturer's instructions (10 min at 95°C, 40 cycles of 15 s at 95°C followed by 1 min at 60°C; dissociation curve 1 min at 95°C, 30 s at 55°C, 30 s at 95°C). Data were analyzed using SA Bioscience PCR Array Data Analysis 3.5 Web Portal (http://dataanalysis.sabiosciences.com/pcr/arrayanalysis.php).

### Data and statistical analysis

Data are presented as the lightest placenta as a percentage of the heaviest in a litter (dotted line = 100%), placentas of P0 fetuses as a percentage of WTL (dotted line = 100%), or median and [range] where the experimental *N* = number of litters, *n* = number of placentas or fetuses. The solid line on graphs represents the median value. For P0 and WTL fetal and placental weights, average fetal weights for each genotype per litter were calculated and data are shown as a mean of these average weights. Data were analyzed by Wilcoxon matched-pairs signed-rank test or Mann Whitney test. *P* < 0.05 was considered statistically significant.

PCR array data were analyzed using SA Bioscience PCR Array Data Analysis 3.5 Web Portal (http://pcrdataanalysis.sabiosciences.com/pcr/arrayanalysis.php). Fold change [2∧(- Delta Delta Ct)] is the normalized gene expression [2∧(- Delta Ct)] in the test sample (lightest placenta or placenta of P0 fetus) divided by the normalized gene expression [2∧(- Delta Ct)] in the control sample (heaviest placenta or placenta of WTL fetus). Fold-change values >1 indicate an up-regulation in expression, and fold-change values < 1 demonstrate a down-regulation (Supplementary Tables 1, 2). Fold regulation values are shown in **Tables 2, 3**.

## Results

### Fetal and placental weights

As expected, lightest placentas from WT mice demonstrated significantly reduced placental weight vs. heaviest placentas at E18.5 (*P* < 0.0001, Table 1). Fetal weight was significantly reduced (*P* < 0.01) and fetal weight: placental weight (F:P) ratio increased (*P* < 0.0001), in fetuses from lightest vs. heaviest WT placentas (Table 1). Mean fetal weight centiles were lower in the lightest compared to the heaviest placenta group (39th vs. 56th centile; *P* < 0.05) but were not considered growth restricted (normal range 10th−90th centile). Consistent with previous studies (19, 40, 41), placentas from P0 fetuses compared to WTL fetuses were lighter, fetal weight was lower and F:P ratio higher at E18.5 (all *P* < 0.0001, Table 1).

**Table 1:** Placental weight, fetal weight and fetal weight:placental weight (F:P) ratio in the lightest and heaviest placental groups of wild-type (WT) mice and in P0 and wild-type littermates (WTL) at embryonic day (E) 18.5.

	**Lightest**	**Heaviest**	**Lightest/** **Heaviest (%)**	***P*-value**
Placental weight (g)	0.068 (0.052–0.080)	0.087 (0.076–0.100)	78.0 (64.0–89.0)	<0.0001
Fetal weight (g)	1.151 (0.924–1.289)	1.191 (1.018–1.441)	94.5 (79.0–108.0)	0.01
F:P ratio	16.8 (12.8–24.0)	14.2 (11.4–16.8)	119.5 (92.0–166.0)	<0.0001
	**P0**	**WTL**	**P0/WTL (%)**	***P*****-value**
Placental weight (g)	0.065 (0.048–0.100)	0.096 (0.050–0.113)	64.0 (58.3–83.1)	<0.0001
Fetal weight (g)	0.977 (0.735–1.218)	1.179 (0.854–1.386)	80.9 (68.1–85.6)	<0.0001
F:P ratio	14.5 (10.91–18.4)	12.6 (10.0–17.1)	110.6 (100.5–146.4)	<0.0001

*Data are median (range) or presented as the lightest placenta as a percentage of the heaviest in a litter (lightest/heaviest (%) column) in WT mice or as the litter average of P0 fetuses as a percentage of the litter average of their WT littermates (P0/WTL (%) column). Data are analyzed by Wilcoxon matched-pairs signed rank test (lightest vs. heaviest; P0 vs. WTL). P < 0.05 was considered statistically significant*.

### PTHrP protein expression

**Figure 1:**
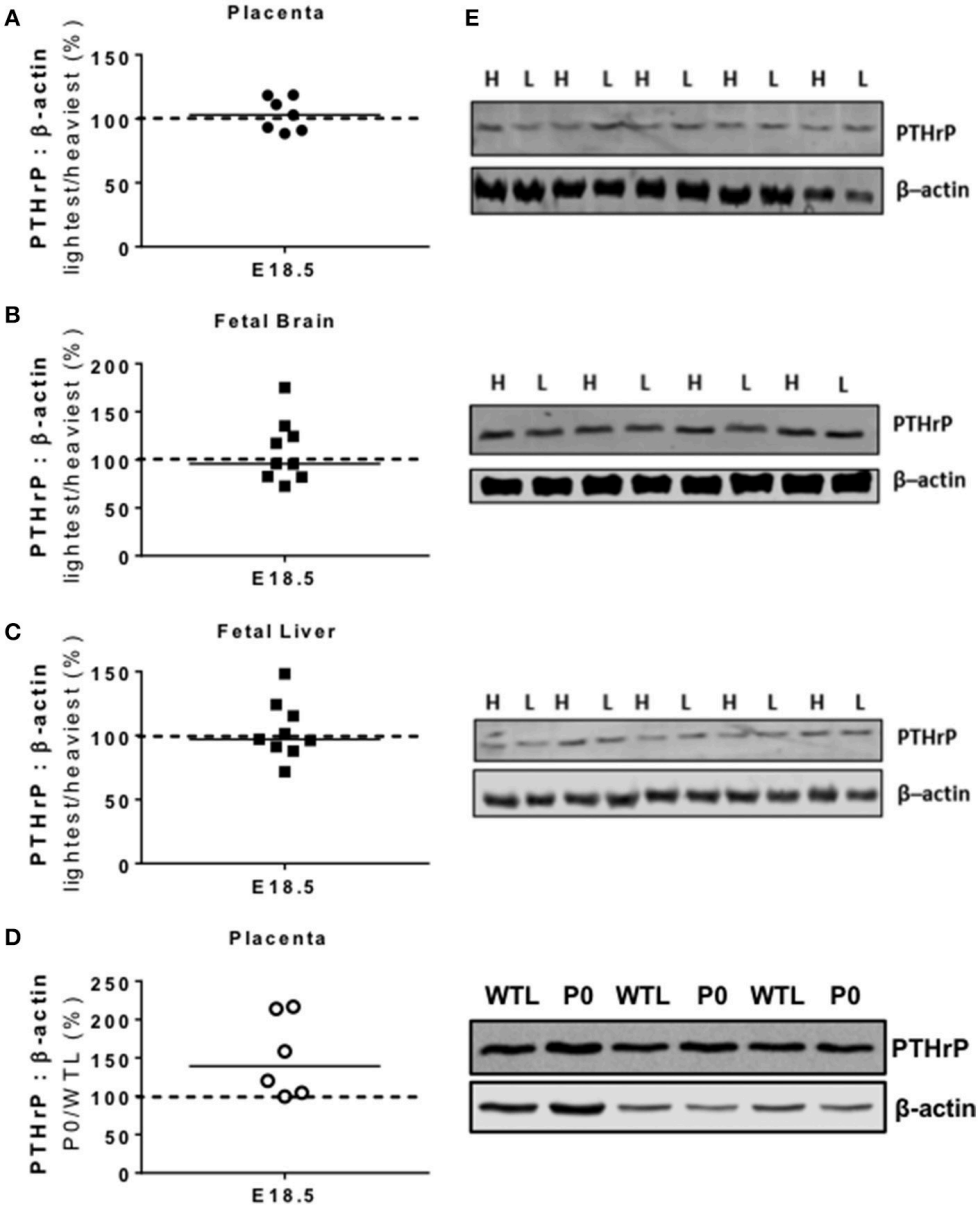
Placental and fetal protein expression of parathyroid hormone-related protein (PTHrP). PTHrP protein expression was not significantly different in paired lightest (L) and heaviest (H) placentas from the same litter **(A)** or in the brains **(B)** and livers **(C)** of the fetuses from these placentas. **(D)** There was a trend for higher PTHrP protein expression in placentas of paired placental-specific insulin-like growth factor 2 knockout (P0) and wild-type (WTL) fetuses (*P* = 0.06) from the same litter. **(E)** Representative Western blots of PTHrP (26 kDa) with the corresponding loading control (β-actin; 42 kDa). Black line = median; dotted line 100% = H or WTL placenta.

There were no significant differences in PTHrP protein expression between the lightest and heaviest placentas of WT mice, or within brains and livers of those fetuses from the lightest and heaviest placentas (Figures 1A–C,E). There was a trend toward increased PTHrP protein expression in placental tissue of P0 vs. WTL fetuses (139%; *P* = 0.06; Figures 1D–E). The PTHrP antibody used for these studies was discontinued during the timecourse of the project and so we were unable to assess PTHrP expression in fetal brains and livers of fetuses in P0 vs. WTL mice. Attempts to use different antibodies targeted to PTHrP failed to show reproducible amplification of signal.

### RT^2^ profiler PCR arrays

**Figure 2:**
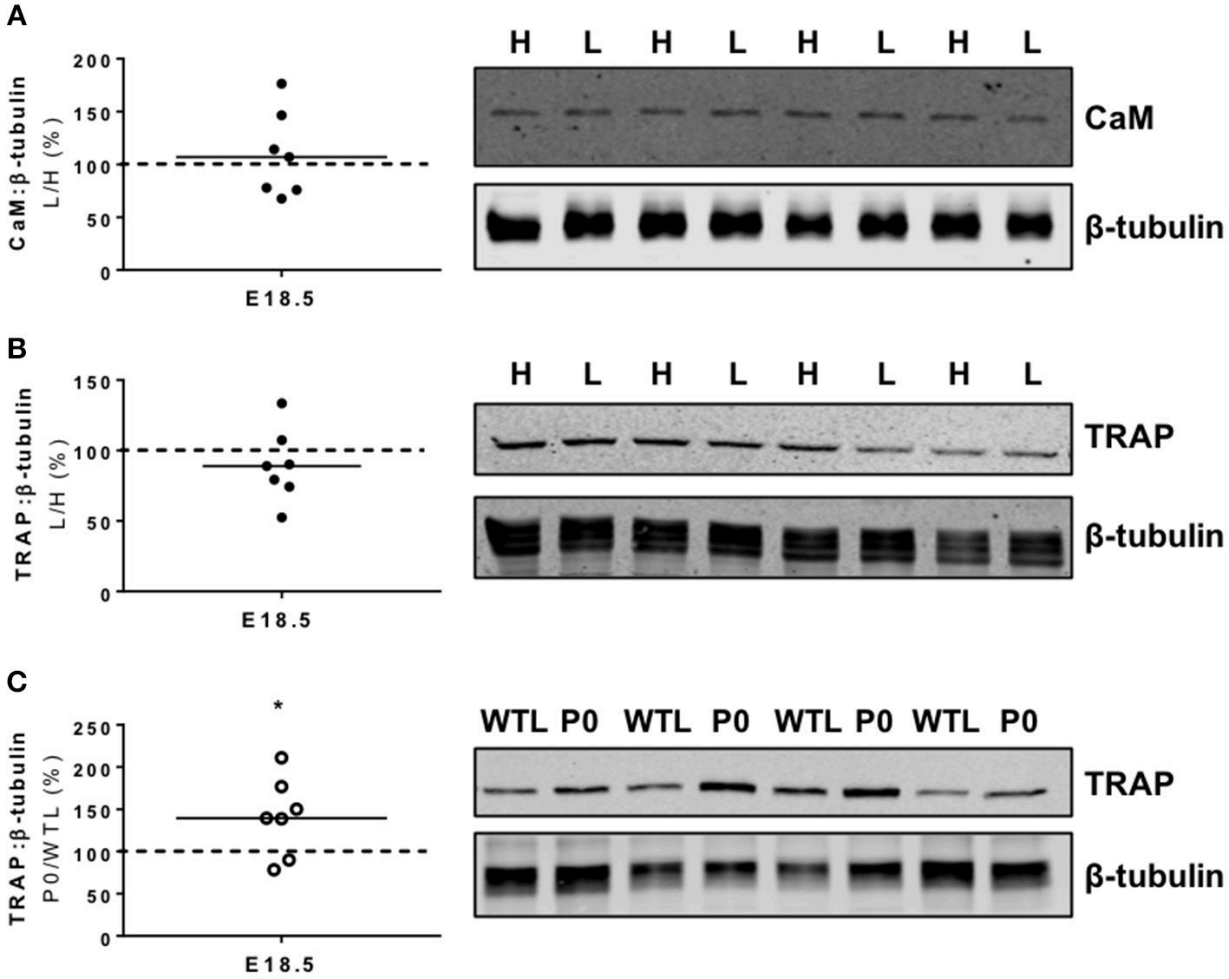
Placental protein expression of calmodulin (CaM) and tartrate resistant acid phosphatase 5 (TRAP). **(A)** CaM and **(B)** TRAP protein expression was not significantly different in paired lightest (L) and heaviest (H) WT placentas from the same litter. **(C)** TRAP protein expression was increased in placentas of placental-specific insulin-like growth factor 2 knockout (P0) compared to wild-type littermates (WTL) from the same litter (*P < 0.05; Wilcoxon signed rank test). Black line = median; dotted line 100% = heaviest WT placenta **(A,B)** or WTL placenta **(C)**. Detected band sizes from representative western blots were as follows; CaM (17 KDa), TRAP (42 kDa) and ß-tubulin loading control (50 kDa).

PCR arrays demonstrated significant changes in the expression of four genes (Table 2); an increase in calmodulin 1 (*Calm1*; *P* < 0.05) and alkaline phosphatase (*Alpl*; *P* < 0.05) expression, and a decreased expression of tartrate resistant acid phosphatase 5 (*Acp5*; *P* < 0.05) and heat shock protein 5 (*Hspa5*; *P* < 0.05) in the lightest vs. heaviest WT placentas (*N* = 6). Placental protein expression of calmodulin (CaM; 107%; Figure 2A) and tartrate resistant acid phosphatase 5 (TRAP; 89%; Figure 2B) measured by Western blot was not different between lightest and heaviest placenta groups.

**Table 2:** Results of the cAMP/Ca^2+^ signaling pathway finder and osteoporosis RT^2^ profiler PCR arrays in the lightest compared to the heaviest placentas from WT mice.

**Gene**	**Product**	**Fold regulation**	***P*-value**
*Calm1*	Calmodulin 1	+1.6	0.04*
*Alpl*	Alkaline Phosphatase	+1.2	0.04*
*Acp5*	Acid Phosphatase 5 Tartrate Resistant	−1.6	0.04*
*Hspa5*	Heat Shock 70 kDa Protein 5	−1.8	0.01*

*Fold regulation is the normalized gene expression in the lightest placentas divided by the normalized gene expression in the heaviest placentas. Fold regulation values +1 indicate an up-regulation in expression, whilst fold regulation values −1 demonstrate a down-regulation*.

* *P < 0.05*.

**Figure 3:**
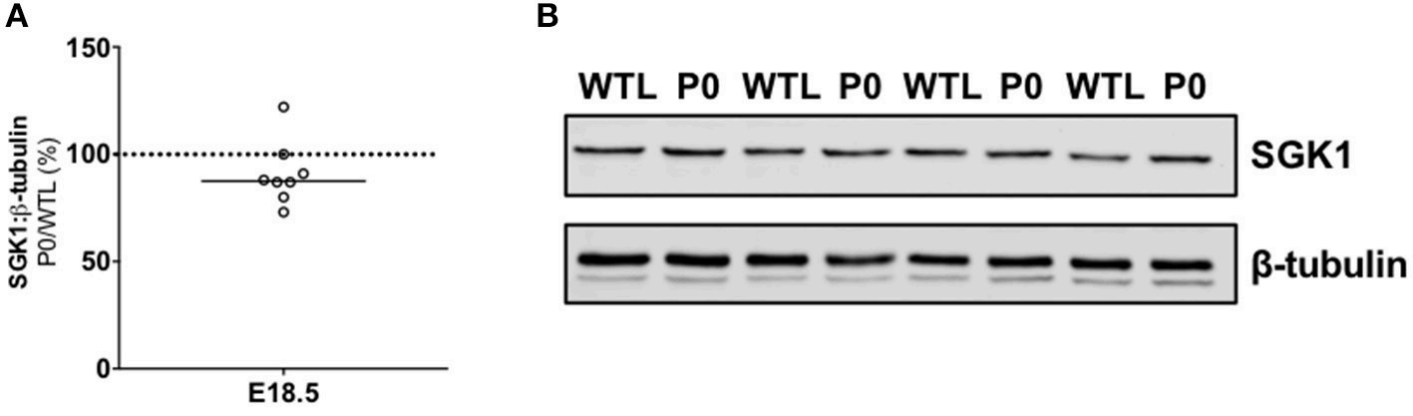
Placental protein expression of serum/glucocorticoid-regulated kinase 1 (SGK1). **(A)** SGK1 protein expression was not significantly different in paired placentas of placental-specific insulin-like growth factor 2 knockout (P0) and wild-type (WTL) fetuses from the same litter. **(B)** Representative Western blots of SGK1 (49 kDa) with the corresponding loading control (β-tubulin; 50 kDa). Black line = median; dotted line 100% = WTL placenta.

Twenty-four genes were differentially expressed in placentas from P0 vs. WTL fetuses in the same litter as shown in Table 3. Of note, significantly reduced expression was observed in the genes encoding calbindin-D_9K_, S100 calcium binding protein G (*S100g*; −1.9-fold, *P* < 0.01), PTHrP (*Pthlh*; −2-fold, *P* < 0.05) and parathyroid hormone 1 receptor (*Pth1r*; −1.7-fold, *P* < 0.01). Expression of serum/glucocorticoid-regulated kinase 1 (*Sgk1*; 1.4-fold, *P* < 0.05), a kinase involved in the regulation of a range of membrane transporters, ion channels and transcription factors as well as cell survival (42–46) was increased. There was no difference in serum/glucocorticoid-regulated kinase 1 (SGK1; 88%; Figure 3) protein expression between placentas of P0 and WT mice.

**Table 3:** Results of the cAMP/Ca^2+^ signaling pathway finder and osteoporosis RT^2^ profiler PCR arrays between placentas from P0 compared to wild-type littermate (WTL) fetuses.

**Gene**	**Product**	**Fold regulation**	***P*-value**
*Ar*	Androgen Receptor	+1.7	0.04*
*Esr1*	Estrogen Receptor	+1.5	0.02*
*Sgk1*	Serum/Glucocorticoid-Regulated Kinase 1	+1.4	0.03*
*Eno2*	Enolase 2, γ Neuronal	+1.4	0.03*
*Tnfrsf11b*	Tumor Necrosis Factor Receptor Superfamily, 11b	+1.4	0.001**
*Tgfb3*	Transforming Growth Factor, β3	−1.2	0.04*
*Rb1*	Retinoblastoma 1	−1.3	0.02*
*Pck2*	Phosphoenolpyruvate Carboxykinase 2 (Mitochondrial)	−1.3	0.04*
*Junb*	Jun-B Oncogene	−1.3	0.04*
*Fosb*	FBJ Osteosarcoma Oncogene B	−1.4	0.02*
*Crem*	cAMP Responsive Element Modulator	−1.4	0.03*
*Per1*	Period Homolog 1 (Drosophila)	−1.4	0.04*
*Nos2*	Nitric Oxide Synthase 2, Inducible	−1.4	0.006**
*Brca1*	Breast Cancer 1	−1.5	0.01*
*Acp5*	Acid Phosphatase 5, Tartrate Resistant	−1.7	0.02*
*Cyp17a1*	Cytochrome P450 Family 17, a1	−1.7	0.03*
*Ltbp2*	Latent Transforming Growth Factor β Binding Protein 2	−1.8	0.03*
*Gem*	GTP Binding Protein (over-expressed in skeletal muscle)	−1.8	0.04*
*S100g*	S100 Calcium Binding Protein G	−1.9	0.004**
*Dkk1*	Dickkopf Homolog 1	−1.9	0.005**
*Wnt10b*	Wingless Related MMTV Integration Site 10b	−1.9	0.04*
*Car2*	Carbonic Anhydrase 2	−2.0	0.009**
*Pthrp*	Parathyroid Hormone-Related Protein	−2.0	0.02*
*Ncam1*	Neural Cell Adhesion Molecule 1	−2.0	0.002**

*Fold regulation is the normalized gene expression in placentas of P0 fetuses divided by the normalized gene expression in placentas of WTL fetuses. Fold regulation values +1 indicate an up-regulation in expression, whilst fold regulation values −1 demonstrate a down-regulation*.

* *P < 0.05*;

** *P < 0.01*.

There was only one similar change in gene expression between the two study groups; tartrate resistant acid phosphatase 5 (*Acp5*, −1.6-and −1.7-fold, respectively) was reduced to the same extent in the lightest placentas of WT mice and placentas of P0 fetuses (Tables 2, 3). Whilst TRAP protein expression was no different between lightest and heaviest WT placentas, gene expression of TRAP was increased in placentas of P0 compared to WT littermates (TRAP; 144%; Figure 2C).

## Discussion

We have previously observed similar adaptive increases in maternofetal calcium transport in small placentas from WT mice, and in small pathological placentas of the P0 knockout mouse model of FGR, with accompanying changes in calbindin-D_9K_ expression (19, 22). Here we demonstrate that the underlying mechanisms of these adaptations in the two models appear to be distinct. Contrary to our hypothesis, expression of PTHrP was not different between lightest compared with heaviest (LvH) WT placentas, but there was a trend toward increased expression in P0 vs. WTL placentas; PTHrP has been shown to influence placental calcium transport in most (32, 33, 36), but not all studies (47). As such, any change in PTHrP expression in placentas near term may be important in the increased maternofetal calcium transfer observed in P0 vs. WTL.

As shown by our previous studies, calbindin-D_9K_ is implicated as a mediator of placental adaptation in calcium transfer both in WT mice (22) and in the P0 mouse (19). However, the lack of change in placental calcium transfer in the calbindin-D_9K_ knockout mouse indicates that other candidates, including calbindin-D_28K_, TRPV5/6 and the sodium-calcium exchanger, might also be involved (48, 49). Thus, we adopted a holistic approach to compare the expression of genes related to calcium transfer and signaling in WT mice and P0 mice. We speculated that these experiments would identify similar calcium-specific pathways altered in the lightest and/or P0 placentas and provide insight into potential regulators of the observed placental adaptation. The number of genes showing altered expression was limited in lightest vs. heaviest WT placentas but mRNA expression of calmodulin-1 was increased in the lightest placentas at E18.5. Whilst this may act as a further indicator of the importance of calcium binding proteins in the previously observed adaptation, this altered expression was not mirrored at the protein level; the importance of the change in gene expression therefore requires further elucidation.

In contrast to the lightest and heaviest placentas in WT mice, multiple genes were differentially expressed in placentas of P0 vs. WT littermates. This is perhaps unsurprising given that P0 mice represent a model of fetal growth restriction whereas lightest vs. heaviest placentas represent extremes of placental weight in a “normal” WT population. Despite the increased maternofetal calcium transport at E18.5, expression of placental calcium-related genes was generally reduced in P0 vs. WTL. Significant reductions were observed in the expression of *S100g* (encoding calbindin-D_9K_), *Pth1r* and *Pthrp*, whilst *Sgk1*, a regulator of epithelial ion transport and cell survival, was up-regulated. SGK1 is a downstream effector of the PI3K/AKT signaling pathway, and in support of the observations here, this pathway is dysregulated in the placentas of P0 knockout mice in late gestation (50). The trend for reduced gene expression near to term could be the result of timing in gestation, i.e., gene expression increased earlier in gestation to promote changes in placental nutrient transport might be downregulated nearer to term having already resulted in increased transcription of the target protein, as previously observed for calbindin-D_9K_ in lightest vs. heaviest WT placentas (22). The choice of E18.5 for these studies reflected the timepoint at which the adaptation (increased placental calcium transport) was previously observed (19, 22) but analyses earlier in gestation would offer further insight into the timing of these changes. With regards to the trend for reduced gene expression near term in P0 vs. WTL, altered gene expression may not be the driving force in these placentas; instead post-translational processing of binding proteins and/or receptors (e.g., TRPV6) may underlie the adaptive changes in calcium transfer. Mechanisms will need to be explored further in future experiments. Whilst the discrepancy in *S100g* gene and calbindin-D_9K_ protein expression at E18.5 will need elucidating, calbindin-D_9K_ does appear to be important in the previously reported changes in placental calcium transport in mouse models of FGR (19, 22).

Increased *Sgk1* expression in P0 vs. WT warrants further investigation to assess whether SGK1, and its activated phosphorylated isoform, play an important role in these adaptive responses by the placenta. SGK1 influences intracellular calcium by up-regulating store operated calcium entry (SOCE), increasing calcium release-activated calcium channel (CRAC) current, and increasing the activity of TRPV5 and 6 (46). Expression and activation of SGK1 is enhanced by higher levels of cytosolic calcium thus SGK1 has been suggested as an amplifier of calcium entry; influx of extracellular calcium through SOCE combined with activation of calcium/calmodulin protein kinase signaling up-regulates levels and activity of SGK1 (42–44). Activation of SGK1 also occurs through other mechanisms, including through the phosphatidylinositol-3-kinase pathway that when stimulated by growth factors activates the mechanistic target of rapamycin complex 2 triggering the phosphorylation of 3-phosphoinositide-dependent kinase PDK1 and subsequent phosphorylation of SGK1 (45, 51). Identifying extracellular regulators stimulating SGK1 intracellular activity may provide potential candidate signals initiating placental adaptations.

Placental *Acp5* gene expression was lower in the lightest compared to the heaviest placentas and in P0 fetuses compared to their WTLs. In contrast, TRAP5 protein expression (encoded by *Acp5*) was not different between the lightest and heaviest placentas, and significantly higher in placentas of P0 compared to WTLs. Previous studies in animal models indicate *Acp5* has an essential role in modeling, remodeling and mineralization of developing bone and cartilage (52), as well as participating in iron transfer from mother to fetus (53). Hansson et al. (54) demonstrated increased placental *Acp5* gene expression in pre-eclampsia compared to normal pregnancy and suggested that this increase might be a compensatory mechanism for poor placentation to prevent fetal malnutrition (54). The elevated expression of TRAP5 protein in placentas of P0 vs. WTL fetuses, which may be as a result of increased gene expression earlier in gestation leading to increased protein translation, supports a regulatory role for TRAP5 in this mouse model of FGR; the added complexity of the opposing direction of change in gene and protein expression, suggest post-translational modification is very important.

For all of the studies described herein, there was an unequal distribution of fetal sex when considering the placental samples analyzed. For the studies comparing lightest vs. heaviest placentas in WT mice, there was a bias toward females having the lightest placentas and males having the heaviest placentas. Whilst we have previously reported that adaptive changes in maternofetal calcium transport do not appear to be influenced by fetal sex (22), future studies investigating the mechanisms underpinning these adaptations should also consider sex-dependent effects. Likewise, fetal sex should be taken into account when assessing mechanisms underpinning placental adaptations in P0 vs. WTL mice.

In summary this study has shown differences in the mechanisms underlying adaptations in placental calcium transport in normal pregnancy vs. that affected by growth restriction. Our data suggest that calcium binding proteins in normal mouse pregnancy, and PTHrP and *Acp5*/TRAP in FGR (P0) pregnancy, are candidate adaptation regulatory proteins worthy of further investigation.

## Author contributions

KM performed research. SG and CS contributed to the conception and design of the work. CH and MD contributed to the conception and design of the work and performed research. All authors were involved in drafting the paper.

### Conflict of interest statement

The authors declare that the research was conducted in the absence of any commercial or financial relationships that could be construed as a potential conflict of interest.
