# Supplementary material for: Mechanisms Underpinning Adaptations in Placental Calcium Transport in Normal Mice and Those With Fetal Growth Restriction
Source: Front Endocrinol (Lausanne). 2018 Nov 20;9:671. doi: 10.3389/fendo.2018.00671 (PMC6255882; doi:10.3389/fendo.2018.00671)
Supplement: Supplementary file 1 [file Table_1.DOCX]

| **Gene ontology** | **Lightest vs Heaviest**  **Fold Change** | | | | | |
| --- | --- | --- | --- | --- | --- | --- |
|  | **<1** | | **1-2** | | | |
| **Serum Responsive Element Enhancer Regulated** | *Junb* |  | *Cnn1* | *Fos* | *Thbs1* | |
|  |  |  | *Cyr61* | *Fosb* | *Vcl* | |
|  |  |  | *Egr1* | *Hspa4* |  | |
|  |  |  | *Egr2* | *Srf* |  | |
| **Calcium Responsive Element Enhancer Regulated**  -Neuropeptides and Neurotransmitters | *Prl* |  | *Adrb1* | *Krtap14* | *Scg2* | |
|  | *Slc18a1* |  | *Cga* | *Nos2* | *Sst* | |
|  |  |  | *Chga* | *Penk* | *Sstr2* | |
|  |  |  | *Gcg* | *S100a8* | *Tacr1* | |
|  |  |  | *Inhba* | *S100a9* | *Th* | |
|  |  |  | *Kcna5* | *S100g* | *Vip* | |
| - Cell Cycle, Cell Survival and DNA Repair | *Nf1* |  | *Bcl2* | *Ccnd1* | *Gem* | |
|  | *Pmaip1* |  | *Brca1* | *Cdk5* | *Pcna* | |
|  | *Ppp1r15a* |  | *Ccna1* | *Cdkn2b* | *Rb1* | |
| - Growth Factors | *Tgfb3* |  | *Areg* | *Crh (21.5 fold change)*  *Fgf6* | | |
|  | *Tnf* |  | *Bdnf* |  |  |  |
| - cAMP Signalling |  |  | *Dusp1* |  | |  |
| - Signal Transduction | ***Hspa5 **** | *Ppp2ca* | *Prkar1a* |  | |  |
|  | *Pln* | *Sgk1* |  |  | |  |
| - Transcription Factors | *Maf* |  | *Atf3* | *Jund* | | *Stat3* |
|  | *Pou2af1* |  | *Creb1* | *Per1* | |  |
|  |  |  | *Crem* | *Pou1f1* | |  |
| - Metabolism | *Ldha* |  | *Ahr* | *Eno2* | | *Pck2* |
|  | *Sod2* |  | *Amd1* | *Hk2* | |  |
| - Immune Regulation | *Mif* |  | *Il2* | *Il6* | | *Ptgs2* |
| **Regulated by Other Calcium Responsive Elements** | *Calb1* | *Ncam1* | *Calb2* | ***Calm1 **** | |  |
|  | *Calr* | *Plat* | *Calcrl* | *Npy* | |  |
|  | *Ddit3* |  |  |  | |  |
|  |  |  |  |  | |  |
| **Bone Remodelling and BMP Signalling** | *Alox15* |  | *Adcy10* | *Ctsk* | | *Nos3* |
|  | *Crtap* |  | *Alox12* | *Enpp1* | | *P2rx7* |
|  | *Timp2* |  | *Alox5* | *Hsd11b1* | | *Plod2* |
|  |  |  | ***Alpl **** | *Igf1* | | *Sfrp1* |
|  |  |  | *Bglap* | *Itga1* | | *Sost* |
|  |  |  | *Bmp2* | *Itgb3* | | *Sparc* |
|  |  |  | *Clcn7* | *Mmp2* | | *Spp1* |
|  |  |  | *Col1a1* | *Mthfr* | | *Stat1* |
|  |  |  | *Col1a2* | *Nfatc1* | | *Twist1* |
|  |  |  | *Comt* | *Nog* | |  |
| **Calciotropic Hormones and Receptors** | *Ar* |  | *Calca* | *Esr1* | | *Pth* |
|  | *Dbp* |  | *Calcr* | *Esr2* | | *Shbg* |
|  | *Pth1r* |  | *Casr* | *Esrra* | | *Tshr* |
|  | *Pthlh* |  | *Cyp17a1* | *Nr3c1* | | *Vdr* |
|  |  |  | *Cyp19a1* | *Prl* | |  |
| **Cytokines, Growth Factors and Receptors** | *Bmp7* |  | *Cd40* | *Il6ra* | | *Npy* |
|  | *Ghrh* |  | *Cnr2* | *Lrp1* | | *Tgfb1* |
|  | *Lepre1* |  | *Fgfr1* | *Lrp5* | | *Tnfaip3* |
|  |  |  | *Fgfr2* | *Lrp6* | | *Tnfrsf1b* |
|  |  |  | *Igfbp2* | *Lta* | | *Vegfa* |
|  |  |  | *Il15* | *Ltbp2* | |  |
|  |  |  | *Il6* | *Mstn* | |  |
| Osteoblast Activity and Differentiation |  |  | *Runx2* |  | |  |
| RANK / RANKL / OPG Signalling |  |  | *Tnfrsf11a* | *Tnfrsf11b* | | *Tnfsf11* |
|  |  |  |  |  | |  |
| **WNT / β-Catenin Signalling** | *Wnt10b* |  | *Dkk1* | *Sfrp4* | | *Wnt3a* |
| **Other Osteoporosis Genes** | ***Acp5 **** | *Car2* | *Lep* | *Mab21l2* | |  |

**Supplementary table 1: Fold change in calcium-related gene expression measured using cAMP/Ca^2+^ signalling pathway finder and osteoporosis RT^2^ profiler PCR arrays in lightest versus heaviest placentas of wild-type mice**. Fold change is the normalized gene expression in the lightest placentas divided by the normalized gene expression in the heaviest placentas. Fold change values >1 indicate an up-regulation in expression, whilst fold-change values <1 demonstrate a down-regulation.*P<0.05 was significant.
