# Supplementary material for: Mechanisms Underpinning Adaptations in Placental Calcium Transport in Normal Mice and Those With Fetal Growth Restriction
Source: Front Endocrinol (Lausanne). 2018 Nov 20;9:671. doi: 10.3389/fendo.2018.00671 (PMC6255882; doi:10.3389/fendo.2018.00671)
Supplement: Supplementary file 2 [file Table_2.DOCX]

| **Gene ontology** | **P0 vs WTL**  **Fold Change** | | | | | |
| --- | --- | --- | --- | --- | --- | --- |
|  | **<1** | | | **1-2**  **(unless stated otherwise)** | | |
| **Serum Responsive Element Enhancer Regulated** | *Cnn1* | ***Fosb **** | ***Junb **** | *Cyr61* |  | *Thbs1* |
|  | *Egr2* | *Hspa4* | *Srf* | *Egr1* |  | *Vcl* |
|  | *Fos* |  |  |  |  |  |
| **Calcium Responsive Element Enhancer Regulated**  -Neuropeptides and Neurotransmitters | *Adrb1* | *Krtap14* | *Slc18a1* | *Inhba* |  |  |
|  | *Cga* | ***Nos2 ***** | *Sst* | *Prl* |  |  |
|  | *Chga* | *Penk* | *Tacr1* | *S100a8* |  |  |
|  | *Gcg* | ***S100g ***** | *Th* | *S100a9* |  |  |
|  | *Kcna5* | *Scg2* | *Vip* | *Sstr2* |  |  |
| - Cell Cycle, Cell Survival and DNA Repair | *Bcl2* | *Cdk5* | *Pmaip1* | *Nf1* |  |  |
|  | ***Brca1 **** | *Cdkn2b* | *Ppp1r15a* |  |  |  |
|  | *Ccna1* | ***Gem **** | ***Rb1 **** |  |  |  |
|  | *Ccnd1* | *Pcna* |  |  |  |  |
| - Growth Factors | *Areg* | *Fgf6* |  | *Bdnf* |  |  |
|  | *Crh* | ***Tgfb3 **** |  | *Tnf* |  |  |
| - cAMP Signalling | *Dusp1* |  |  |  |  |  |
| - Signal Transduction | *Hspa5* | *Ppp2ca* |  | ***Sgk1 **** |  |  |
|  | *Pln* | *Prkar1a* |  |  |  |  |
| - Transcription Factors | *Atf3* | *Jund* | *Pou2af1* | *Maf* |  |  |
|  | *Creb1* | ***Per1 **** | *Stat3* |  |  |  |
|  | ***Crem **** | *Pou1f1* |  |  |  |  |
| - Metabolism | *Amd1* | *Ldha* | *Sod2* | *Ahr* |  |  |
|  | *Hk2* | ***Pck2 **** |  | ***Eno2 **** |  |  |
| - Immune Regulation | *Il2* | *Mif* |  |  |  |  |
|  | *Il6* | *Ptgs2* |  |  |  |  |
| **Regulated by Other Calcium Responsive Elements** | *Calb1* | *Calr* | *Plat* | *Calcrl* |  |  |
|  | *Calb2* | *Ddit3* |  | *Npy* |  |  |
|  | *Calm1* | ***Ncam1 ***** |  |  |  |  |
|  |  |  |  |  |  |  |
| **Bone Remodelling and BMP Signalling** | *Adcy10* | *Col1a2* | *Nfatc1* | *Alox15* | *Itga1* | *Plod2* |
|  | *Alox12* | *Comt* | *Nog* | *Alox5* | *Itgb3* | *Sfrp1* |
|  | *Alpl* | *Crtap* | *Sost* | *Bmp2* | *Mmp2* | *Spp1* |
|  | *Bglap* | *Hsd11b1* | *Sparc* | *Ctsk* | *Nos3* | *Stat1* |
|  | *Clcn7* | *Igf1* | *Timp2* | *Enpp1* | *P2rx7* | *Twist1* |
|  | *Col1a1* | *Mthfr* |  |  |  |  |
| **Calciotropic Hormones and Receptors** | *Calcr* | *Esr2* | *Pth* | ***Ar **** |  |  |
|  | *Casr* | *Esrra* | ***Pth1r ***** | *Calca* |  |  |
|  | ***Cyp17a1 **** | *Nr3c1* | ***Pthlh **** | ***Esr1 **** |  |  |
|  | *Cyp19a1* | *Prl* | *Shbg* |  |  |  |
|  | *Dbp* |  | *Tshr* |  |  |  |
| **Cytokines, Growth Factors and Receptors** | *Fgfr1* | *Il6* | ***Ltbp2 **** | *Bmp7* | *Tgfb1* |  |
|  | *Fgfr2* | *Lrp1* | *Mstn* | *Cd40* | *Tnfaip3* |  |
|  | *Ghrh* | *Lrp5* | *Lepre1* | *Cnr2* | *Tnfrsf1b* |  |
|  | *Igfbp2* | *Lrp6* | *Vegfa* | *Il6ra* |  |  |
|  | *Il15* | *Lta* |  | *Npy* |  |  |
| Osteoblast Differentiation |  |  |  | *Runx2* |  |  |
| RANK / RANKL / OPG Signalling | *Tnfsf11* |  |  | *Tnfrsf11a* |  |  |
|  |  |  |  | ***Tnfrsf11b ***** |  |  |
| **WNT / β-Catenin Signalling** | ***Dkk1 ***** | *Wnt3a* |  | *Sfrp4 (2.4 fold change)* |  |  |
| **Other Osteoporosis Genes** | ***Acp5 **** | *Lep* |  |  |  |  |
|  | ***Car2 ***** | *Mab21l2* |  |  |  |  |

**Supplementary table 2: Fold change in calcium-related gene expression measured using cAMP/Ca^2+^ signalling pathway finder and osteoporosis RT^2^ profiler PCR arrays in placentas of P0 versus wild-type littermates (WTL)**. Fold change is the normalized gene expression in placentas of P0 fetuses divided by the normalized gene expression in placentas of WTL fetuses. Fold change values >1 indicate an up-regulation in expression, whilst fold-change values <1 demonstrate a down-regulation.*P<0.05, **P<0.01.
